# Estrogenicity of Glabridin in Ishikawa Cells

**DOI:** 10.1371/journal.pone.0121382

**Published:** 2015-03-27

**Authors:** Melissa Su Wei Poh, Phelim Voon Chen Yong, Navaratnam Viseswaran, Yoke Yin Chia

**Affiliations:** 1 School of Biosciences, Division of Medicine, Pharmacy and Health Sciences, Taylor’s University, No. 1, Jln Taylor’s, 47500 Subang Jaya, Malaysia; 2 Postgraduates, Research and Strategic Development, Taylor’s University, No. 1, Jln Taylor’s, 47500 Subang Jaya, Malaysia; Michigan State University, UNITED STATES

## Abstract

Glabridin is an isoflavan from licorice root, which is a common component of herbal remedies used for treatment of menopausal symptoms. Past studies have shown that glabridin resulted in favorable outcome similar to 17β-estradiol (17β-E_2_), suggesting a possible role as an estrogen replacement therapy (ERT). This study aims to evaluate the estrogenic effect of glabridin in an *in-vitro* endometrial cell line -Ishikawa cells via alkaline phosphatase (ALP) assay and ER-α-SRC-1-co-activator assay. Its effect on cell proliferation was also evaluated using Thiazoyl blue tetrazolium bromide (MTT) assay. The results showed that glabridin activated the ER-α-SRC-1-co-activator complex and displayed a dose-dependent increase in estrogenic activity supporting its use as an ERT. However, glabridin also induced an increase in cell proliferation. When glabridin was treated together with 17β-E_2_, synergistic estrogenic effect was observed with a slight decrease in cell proliferation as compared to treatment by 17β-E_2_ alone. This suggest that the combination might be better suited for providing high estrogenic effects with lower incidences of endometrial cancer that is associated with 17β-E_2_.

## Introduction

Two of the trials by the Women's Health Initiative (WHI) on women who received estrogen replacement therapy (ERT) had ended early due to greater risks in heart disease, breast cancer and stroke which outweighed the benefits of decrease in risk of fractures. Since the release of the results, many women have stopped their ERT regimen and turned towards natural plant compounds as alternative instead [1, 2]. Licorice root of the glycyrrhiza plant has been widely used in Asia [3] as a herbal medicine [4]. They are a rich source of flavonoids [5] which may serve as natural estrogen agonists in preventing the symptoms and diseases associated with estrogen deficiency [6]. Thus they might offer opportunities for its development into supplements aimed at women with menopausal symptoms. Previous studies have shown that licorice root extracts have estrogenic activity towards estrogen receptors [7]. This estrogenicity of licorice had been attributed to the presence of phytoestrogens such as glabridin, the main isoflavan present in licorice root [6, 8].

Both glabridin and 17β-estradiol (17β-E_2_) have three fused rings of phenanthrene shape and an aromatic ring substituted with hydroxyl group at the para (glabridin) or 3 position (17β-E_2_). Both molecules contain second hydroxyl group (17β in 17β-E_2_ and 29 in glabridin) and is therefore relatively lipophilic [6]. This similarity suggests that glabridin could possibly bind to the same receptors to 17β-E_2_ to to bring about estrogenic activity.

Based on past literatures, glabridin had exhibited estrogenic effect similar to that of 17β-E_2_ in bone, breast and heart models [8, 9]. This suggests that glabridin could have estrogenic activity similar to that of estrogen replacement therapy (ERT) agent. Therefore, glabridin can be further explored for estrogenic activity in endometrial tissues, which is the site where majority if the effects take place for women taking ERT. This study aims to elucidate the estrogenicity of glabridin compared to that of 17β-E_2_. Alkaline phosphatase (ALP) assay was used to test for estrogenic activity and the ER-α-ligand-co-activator activity assay was performed to investigate on the agonistic or antagonistic activity of glabridin at ER-α. Meanwhile, MTT assay was used to evaluate the effects of glabridin on cell proliferation. This study also compared for the first time, the estrogenic activity of glabridin with other estrogens such as estrone (E_1_), ethinyl estradiol (EE) and Premarin. In addition, the combinatorial effect of glabridin with 17β-E_2_ was also evaluated and any synergistic activity was determined using the synergy quotient calculation.

## Materials and Methods

### Chemicals

Minimum Essential Medium (MEM), 17β-E_2_, E_1_, EE and *p*-nitrophenyl phosphate (pNPP), Dimethyl sulfoxide (DMSO) and dextran-coated charcoal (DCC) were purchased from Sigma, USA. Fetal bovine serum (FBS), fetal calf serum (FCS), antibiotic-antimycotic and L-glutamine were obtained from Gibco, USA. Dulbecco's Modified Eagle Medium: Nutrient Mixture F-12 (DMEM/F-12) was sourced from Nacalai Tesque, Japan. Glabridin was purchased from Wako, Japan. Phosphate buffered saline (PBS) was from OXOID, USA. Thiazoyl blue tetrazolium bromide (MTT) was obtained from Amresco, USA. Premarin was obtained from Wyeth Pharmaceuticals, USA. Receptor cofactor assay (RCAS) was purchased from Cosmo Bio Co., Ltd. All other chemicals were purchased from Merck, Germany.

### Cell culture and maintenance of Ishikawa cells

Ishikawa cells (99040201, ECACC), which are human endometrial adenocarcinoma cells, were maintained in MEM supplemented with 10% FBS, 1% antibiotic-antimycotic and 1% L-glutamine. Cells were passaged twice weekly. Two days before the start of the experiment, near-confluent cells were changed to an estrogen-free basal medium (EFBM). The EFBM consist of DMEM/ F-12 media supplemented with 5% DCC-stripped FCS, 1% antibiotic-antimycotic and 1% L-glutamine. After 24 h, the cells were harvested with 0.25% EDTA-trypsin and seeded in 96-well flat-bottomed microtiter plates, in 100 μl of EFBM/ well.

### Preparation of test compounds

All the test compounds were dissolved in ethanol (EtOH) and aliquoted accordingly. Prior to use, the test compounds were diluted appropriately with EFBM until the final concentration of EtOH was at 0.1% (v/v). All test compounds were stored at −20°C.

### Alkaline Phosphatase Activity Assay

For this study, the ALP was used as the main assay in evaluating the estrogenicity of glabridin in Ishikawa cells, as described previously [10]. Briefly, Ishikawa cells were seeded at a density of 1.5x10^4^cells/100 μL in EFBM/well in a 96-well flat bottomed plate. After incubation with test compounds for 72 h, 50μL of ice-cold pNPP solution was added and read at 405nm. All experimental conditions were assayed in triplicate.

### Receptor Cofactor Assay

Receptor cofactor assay (RCAS) by Cosmo Bio Co., Ltd was used to determine the ER-α-ligand-co-activator activity of the glabridin. Competitive ER-α binding assay only determines if a ligand binds to ER-α, whereas RCAS detects ER-α-ligand-co-activator complex as per manufacturer’s protocol. The detection of ER-α-ligand-co-activator enables a more accurate prediction of whether a ligand acts as an agonist or antagonist. Briefly, a peptide containing LXXLL motif of co-activator (SRC-1) was immobilized on the 96-well plate, followed by addition of the compounds to be tested and ER-α. Horseradish peroxidase (HRP) conjugated antibody solution was then added, followed by tetramethylbenzidine (TMB) substrate. Finally, stop solution was added and the plate was read at 450nm via the BIORAD iMark Microplate Absorbance Reader. The activity of 1nM 17β-E_2_ on the ER-α-SRC-1-co-activator complex was treated as 100% and the activity of all other treatments were compared to it. All experimental conditions were assayed in triplicate.

### Combination study

In the combinatorial studies, the checkerboard dilution method was used. The concentration of the drugs prepared were doubled and added in a ratio of 1:1 to the cells. The combination study was performed to screen the drugs for any antagonist, additive agonist or synergistic estrogenic effects when treated together as compared to their estrogenic effects when treated individually.

### Synergy quotient calculation for synergism

The synergism quotient (SQ) was calculated by subtracting baseline values from all treatments and then dividing effects of combined treatments by the sum of individual treatments. A SQ greater than 1.0 indicates that there is synergism for a given measured response [11, 12].

### MTT Cell proliferation assay

The effect of the extracts on cell proliferation were estimated using MTT assay, as per previously described [10]. Briefly, 10μL of 5mg/ml MTT was added to each well after incubation with test compounds for 72 h. DMSO was then added to dissolve the formazan and the plate read at 595nm. The cell proliferation of control cells was 100.00% and any increase or decrease in cell proliferation of treated cells was compared to the control cells. All experimental conditions were assayed in triplicate.

### Statistical analysis

All data were be expressed as mean + SEM (Standard Error of Mean). Data collected were analyzed using student’s t-test via a statistical software SPSS version 16.0 (SPSS Inc., USA), where * P<0.05 and ** P≤0.01.

## Results and Discussion

### Glabridin induced dose-dependent increase in estrogenic activity and cell proliferation in Ishikawa cells

**Fig 1:**
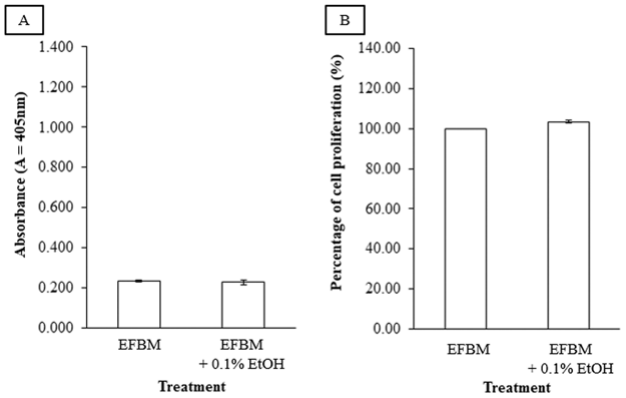
Comparison of A) ALP and B) cell proliferation activity of Isihkawa cells treated with EFBM only versus EFBM + 0.1% EtOH (untreated control). The values represent the mean activity of three treatment group. There was no significant difference between the two different treatments.

ALP assay had been used as the main experimental design for accessing estrogenic effect while MTT assay had been used to study cell proliferation. Ishikawa cells represents a suitable cell model for this study as it responds to estrogens at concentrations approximating physiological levels [13]. As the drugs used in this study were dissolved in EtOH which was diluted down to a concentration of 0.1% before treatment on to the cells, the effect of EFBM with 0.1% EtOH on the ALP activity of Ishikawa cells was tested. Fig. 1A showed that there was no significance difference in the ALP activity induced in cells treated with the EFBM alone and those treated with EFBM containing 0.1% EtOH. Hence, any effect on ALP activity was deduced to be due to the effect of the drugs and not EtOH. As with the ALP assay, the effect of EtOH as a carrier for the drugs was tested for its effect on cell proliferation in Ishikawa cells. The result showed that there was no significance difference between the EFBM only and EFBM with 0.1% EtOH, as shown in Fig. 1B.

**Fig 2:**
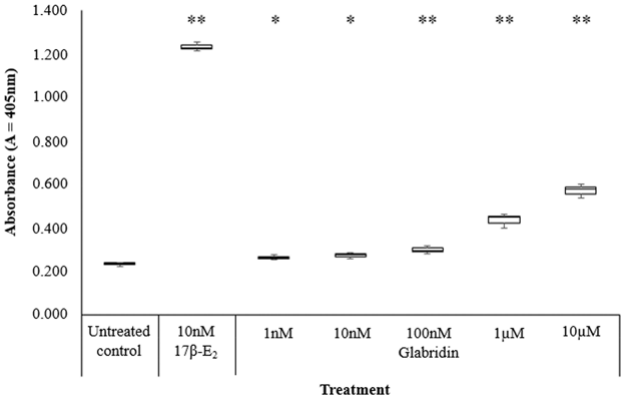
Effect of varying concentrations of glabridin on ALP activity in Ishikawa cells in comparison to the untreated control and 1nM 17β-E_2_. The values represent the mean activity of three treatment group. *P<0.05 and ** P≤0.01 against the untreated control.

In line with our previous study, the stimulation of ALP activity by 17β-E_2_ was dose dependent and maximum response was at 10nM 17β-E_2_ (1.22 ± 0.00A) which was 5-fold of that induced by the untreated control (0.23 ± 0.01A) [14–16]. In this study, it was found that as with 17β-E_2_, glabridin induced dose dependent increase in ALP activity from 100pM to 10μM [15]. The maximum induction was observed at 10μM (0.61 ± 0.02A), which was only half of the induction of the positive control- 17β-E_2_ at 1nM (1.10 ± 0.03A) (Fig. 2). However, as with other phytoestrogens such as genistein and daidzein, highest ALP activity was observed at 10μM, which is 2–4 fold compared to the control [17, 18]. For glabridin, the EC_50_ of glabridin was observed to be at 780nM.

### Synergistic activity of glabridin in the combination treatment

**Fig 3:**
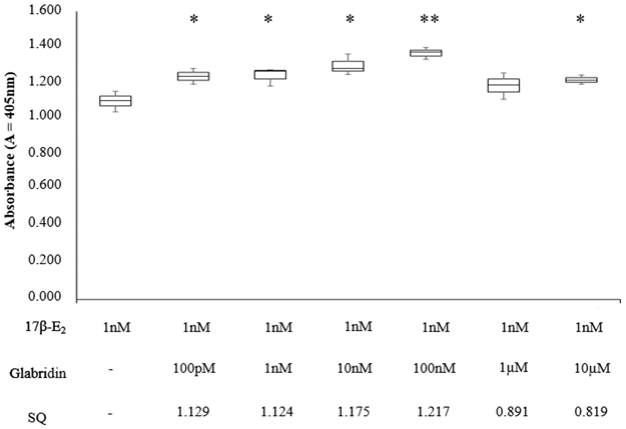
Effect of varying concentrations of glabridin with 1nM 17β-E_2_ on ALP activity in Ishikawa cells in comparison to 1nM 17β-E_2_. SQ values were calculated to determine if there was any synergistic activity in the combination treatment. The values represent the mean activity of three treatment group. *P<0.05 and ** P≤0.01, as compared to 1nM 17β-E_2_.

Synergy assessment has become a key area in phytomedicine research in recent years as therapeutic superiority is often observed for many multidrug combinations in traditional medicine over single constituents [19]. Hence in this study, the co-effect of glabridin with 17β-E_2_ was also evaluated. For the combination study, synergistic activity was observed between 100pM — 100nM glabridin and 1nM 17β-E_2_, calculated based on the SQ calculation (Fig. 3). The maximum estrogenic activity was observed at 100nM glabridin and 1nM 17β-E_2_, with a SQ of 1.217.

In a separate combinatorial study of isoflavones with 17β-E_2_ performed by Kayisli *et al*. [15] via ALP assay in Ishikawa cells, the results showed that when treated alone, the isoflavones induced a weak estrogenic activity (39–67% less than 17β-E_2_); but when treated with 17β-E_2_, they antagonized estrogen induced alkaline phosphatase activity by 36–89%. The result of this study differed from that as while glabridin too acts as a weak agonist alone it does not act as an estrogen antagonist in the presence of 17β-E_2_. However, this could be due to the difference in treatment method in both studies. In this study, both drugs were treated together at the same time. However, Kayisli *et al*. [15] treated the Ishikawa cells with 10μM isoflavones 30minutes before 17β-E_2_ treatment. In doing so, they observed a significant decrease in ALP activity in a time-dependent manner (36–89%). However, when the cells were treated with isoflavones and 17β-E_2_ simultaneously, the isoflavones were less effective in decreasing the ALP activity. Hence, it seems that the sequence in which the drugs were treated had an effect on ALP activity.

Previously, glabridin had also been shown to act as an estrogen agonist in uterus of rats in *in-vivo* assays [6] by binding to estrogen receptors (ERs). While no other studies have evaluated the combinatorial effect of glabridin with 17β-E_2_, Harris *et al*. [20] had shown that genistein and daidzein when treated against a 0.5nM 17β-E_2_ background in MCF-7 breast cancer cells, displayed additive agonist luciferase activity. The investigators suggested that this was because the MCF-7 luciferase assay was not maximized by the highest dose of 17β-E_2._ However, the mechanism for this effect is unknown.

In this study, it could be observed that while a lower concentration of glabridin with 1nM 17β-E_2_ induced synergistic activity, at higher concentrations of glabridin (1μM and 10μM), an antagonistic activity was observed. This could be because glabridin exerts a lower activity at ERs in Ishikawa cells compared to 1nM 17β-E_2_. At high glabridin concentrations, more glabridin is present to compete with 17β-E_2_ for binding to ERs and hence result in a lower ALP response. The increase in ALP activity observed in Ishikawa cells due to the combination drug treatment could be due to the ALP assay not being maximized by the highest dose of 17β-E_2._ It had also been suggested that as the binding affinities of phytoestrogens to the ERs are only a fraction of that of 17β-E_2_ (especially at estrogen receptor-α (ER- α)) [21], the super-agonist activity may be due to extra-genomic actions [20]. This super-agonist effect has also been observed previously in transfected MCF-7 cells treated with genistein and daidzein using luciferase assay [20].

### Glabridin exerts estrogenic activity via the ER-α-SRC-1-co-activator complex

In the past, Tamir et al. [6] showed that glabridin displayed ER binding activity. In a separate study, Simons et al. [5] showed that glabridin displayed no significant activity on ER at 100nM to 100µM. Meanwhile, ethyl acetate glabridin–rich fractions from *glycyrrhiza glabra* displayed ER-α-selective antagonism when tested for estrogen activity using yeast estrogen bioassays [5]. These ER binding assay [5, 6, 22] only demonstrates the ligand-binding ability of a compound. Thus, a more specific evaluation such as the receptor cofactor assay is required to analyze if the activation occurs via the ER-α-SRC-1-co-activator. However, the specific estrogenic potencies of glabridin towards ER-α via SRC-1 have not yet been investigated. Such information is vital for understanding their specific estrogenic activity.

**Fig 4:**
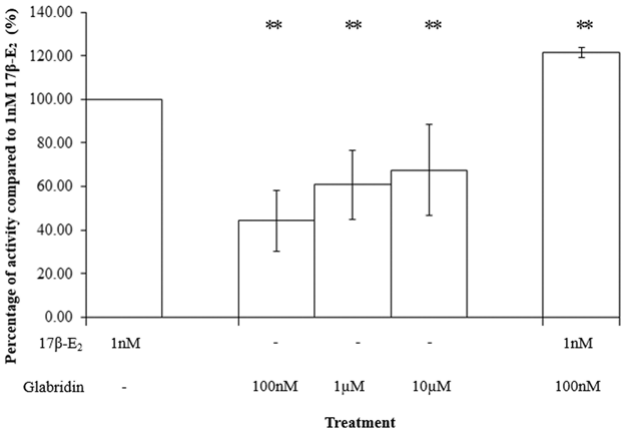
Comparison of the activity of the glabridin, 17β-E_2_ and the combination treatment of glabridin and 17β-E_2_ to 1nM 17β-E_2_ in the ER-α-SRC-1-co-activator complex. The values represent the mean activity of three treatment group. *P<0.05 and ** P≤0.01, as compared to 1nM 17β-E_2_.

The result showed that glabridin exerted a dose-dependent increase in ER-α-SRC-1-co-activator activity (Fig. 4). For glabridin, the activity via the ER-α-SRC-1-co-activator complex was almost half of 1nM 17β-E_2_. As glabridin had a lower activity compared to 17β-E_2_, this suggests that glabridin displayed partial agonist activity at ER-α-SRC-1-co-activator complex.

When 100nM of glabridin was tested together with 1nM 17β-E_2_, there was a significant increase of 21.4 ± 2.21% in ER-α-SRC-1-co-activator complex activity as compared to 1nM 17β-E_2_. This showed that in the presence of 17β-E_2_, glabridin did not display antagonist effect at the ER-α-SRC-1-co-activator complex. In the past, many studies have made use of ALP as a marker of ER activation and ER-dependent differentiation in Ishikawa cells [23–27]. Together with the ALP assay, the ER-α-SRC-1-co-activator assay showed that glabridin exerted estrogenic activity via the ER-α-SRC-1-co-activator complex. Similarly, the increase in activity in the combination treatment could have occurred via the ER-α-SRC-1-co-activator complex.

### Glabridin induced dose-dependent increase in cell proliferation in Ishikawa cells

**Fig 5:**
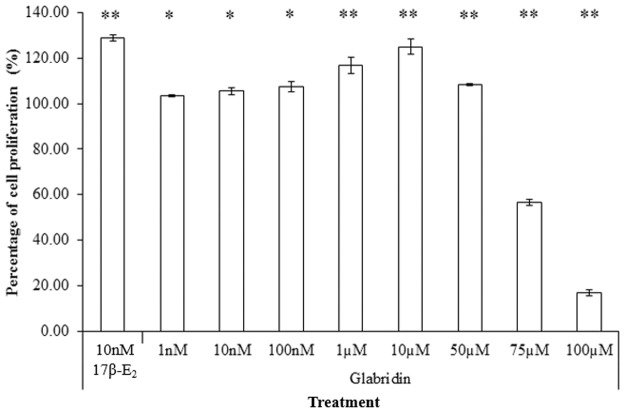
Effect of varying concentrations of glabridin in comparison to 1nM 17β-E_2_. The values represent the mean activity of three treatment group. *P<0.05 and ** P≤0.01 against the untreated control.

Based on the MTT assay, glabridin also induced a dose-dependent increase in cell proliferation from 1nM to 10μM, similar to that of 17β-E_2_ [16], though not as high as 10nM 17β-E_2_ (Fig. 5). This increase in cell proliferation was also observed in other phytoestrogens such as genistein [28]. For glabridin, the maximum induction of cell proliferation corresponds to the highest ALP induction concentration of 10μM glabridin (22.37% increase from control). This suggests that ALP activity and cell viability in Ishikawa cells induced by glabridin are closely associated, whereby an increase in ALP activity seems to be followed by an increase in cell proliferation.

Glabridin at concentrations higher than 10μM was toxic to Ishikawa cells and decreased cell proliferation, There was 83.14 ± 1.32% of cell death occurring at 100μM. This biphasic effect demonstrated by glabridin in Ishikawa cells suggested that they regulate estrogenic activity and cell proliferation not only through ER in a dose-dependent manner but also via ER-independent mechanism [6]. The results observed was similar to a separate study by Simons *et al*. [5] whereby glabridin was also shown to be toxic to yeast cells at 100μM and above. In this study, the IC_50_ of glabridin was observed to be at 60μM. Several past studies using various mammalian proliferation assays have similarly reported the IC_50_ of glabridin to be at around 5μM [6, 8, 22].

**Fig 6:**
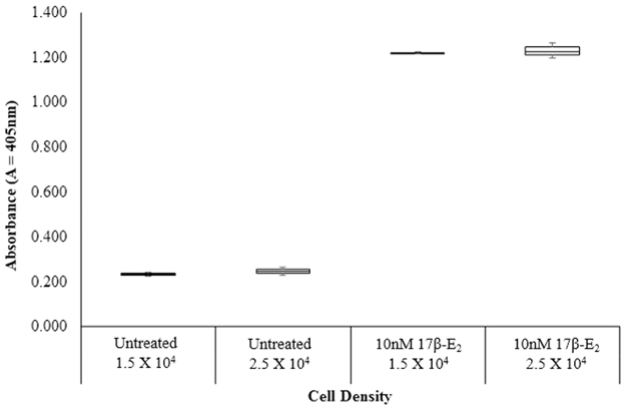
Effect of varying seeding density on the effect of 17β-E_2_ on the induction of cell proliferation in Ishikawa cells in comparison to the untreated control. The values represent the mean activity of three treatment group.

Due to the similarity in the trend of 17β-E_2_ and glabridin’s effect on ALP and MTT, it might be suggested that ALP activity and cell viability in Ishikawa cells induced by 17β-E_2_ were closely associated. Hence, the effect of both high (2.5 x 10^4^ cells) and low (1.5 x 10^4^ cells) cell density on the ALP induction were tested and the results were as shown in Fig. 6. The result showed that there were no significant differences between high and low densities for the untreated control and those treated with 10nM 17β-E_2_. The results observed in this study was similar to past studies which showed that the starting cell densities of cells do not greatly affect ALP induction in Ishikawa cells. As reported in 1986 by Holinka *et al*., any increase in ALP activity is due to stimulation by 17β-E_2_ and not due to increase in cell numbers because ALP is an estrogen-inducible gene at transcriptional level [14].

### Glabridin had little effect on the cell proliferative effect of 17β-E_2_ in combination treatment

**Fig 7:**
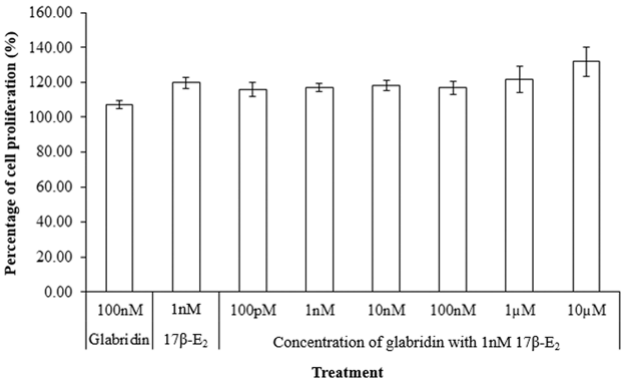
Effect of varying concentrations of glabridin with 1nM 17β-E_2_ on the induction of cell proliferation in Ishikawa cells in comparison to 1nM 17β-E_2_. There was no significant difference between the combination treatments with 1nM 17β-E_2_.

A look at the effect of glabridin against a background of 1nM 17β-E_2_ showed that there was no significant increase in cell proliferation as compared to 1nM 17β-E_2_ (Fig. 7). In the past, Tamir *et al*. [6] showed that glabridin did not have much effect on the cell proliferative effect of 17β-E_2_ in breast cancer cells. Likewise, this study observed that glabridin (100pM to 10μM) had little influence on the effect of 17β-E_2_ on cell proliferation in Ishikawa cells with a maximal difference of less than 20% with the addition of glabridin at certain concentrations. However, there was still a slight decrease in cell proliferation in the combination treatment of 100nM glabridin with 1nM 17β-E_2_ (16.98 ± 3.76% increase from control) compared (19.67 ± 3.42% increase) to 1nM 17β-E_2_, though not significant. Meanwhile, the combination did show a significant decrease in cell proliferation from that of 10nM 17β-E_2_ (28.79 ± 1.26% increase). Both the ALP and RCAS assay showed that 100nM glabridin with 1nM 17β-E_2_ significantly increased ALP and ER-α-SRC-1-co-activator activity compared to 1nM 17β-E_2_ but a decrease in induction of cell proliferation was observed compared to treatment with 1nM 17β-E_2_ only. It is unknown why there was a decrease, but perhaps ER-α-independent actions might be involved.

The result observed was similar to several past studies whereby treatment of the biological sample with the drug compound resulted in an increase in cell proliferation but when treated in high concentration or with 17β-E_2_ decreased cell proliferation. According to Harris *et al*. [20], the mechanisms for the decrease in cell proliferation is unclear. One hypothesis proposed is that the presence of low-affinity or low-potency ligands for ERs can reduce the effect of potent endogenous estrogens such as 17β-E_2_ when they are present in sufficient quantities, with the net effect of antagonizing the estrogen-responsive system. Likewise, in a separate study, Hu *et al*. [29] investigated the effect of DMSO licorice root extract on MCF-7 cell growth and the effect was biphasic. At 10 to 100μg/mL, the extract stimulated growth with maximum effect at about 100μg/mL. However, at concentrations higher than 100μg/mL or when treated with a background of 1nM 17β-E_2_, an inhibition in cell growth was observed.

### Comparison of the estrogenic of glabridin to other estrogens

There are no past study that simultaneously compared the estrogenicity of glabridin with other estrogens such as E_1_, EE and Premarin on an endometrial cell line via ALP assay. E_1_ is the dominant form of estrogen present in menopausal women [30]. EE is a common component of combined oral contraceptives. It consists of an ethinyl group at carbon 17 of ring D of the steroid nucleus which slows down both hepatic and enzymatic degradation. Hence, EE is one of the most potent oestrogens, at 15 to 20 times more active than oral 17β-E_2_ [31]. Lastly, Premarin (manufactured by Wyeth Pharmaceuticals) is an ERT made up of conjugated equine estrogens (CEE) such as equilin sulfate and estrone sulphate, extracted from the urine of pregnant mares [32].

**Fig 8:**
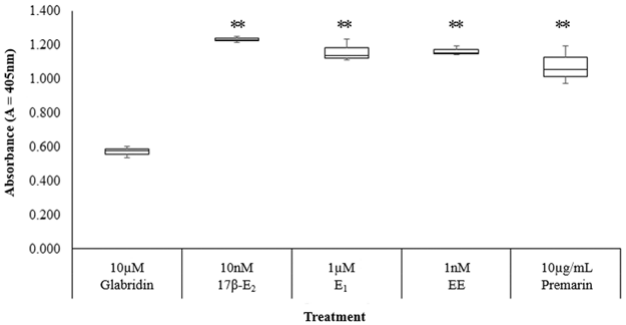
Effect of 10μM glabridin in comparison to 10nM 17β-E_2_, 1μM E_1_, 1nM EE and 10μg/mL Premarin on ALP activity in Ishikawa cells. The values represent the mean activity of three treatment group. *P<0.05 and ** P≤0.01, as compared to the 10μM glabridin.

**Fig 9:**
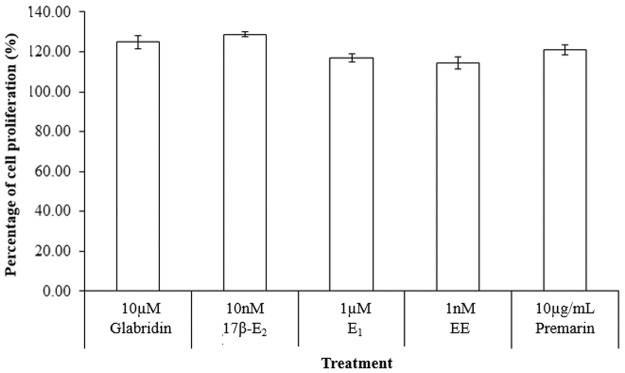
Effect of 10μM glabridin in comparison to 10nM 17β-E_2_, 1μM E_1_, 1nM EE and 10μg/mL Premarin on the induction of cell proliferation in Ishikawa cells. The values represent the mean activity of three treatment group. *P<0.05 and ** P≤0.01, as compared to the untreated control. There was no significant difference between the different treatments with 10μM glabridin.

As with Le Guevel and Pakdel (2001) who tested the induction of estrogenic chemicals on the Lac Z reporter gene in rainbow trout ER recombinant yeast, the ALP activity showed that the estrogens differed in potency: EE> 17β-E_2_> E_1_ > Glabridin, as determined by the amount of drug required to reach the maximal point of induction for ALP activity, Fig. 8. EE induced ALP activity to a maximum at 1nM, which was the lowest amount of drug required to reach such a high level of ALP activity among the drugs tested. Glabridin represented the least potent drug as it requires a high amount of 10μM to reach about half the maximal point of that of other drugs. The result showed that 10μM glabridin achieved only about half maximal activity of all the other drugs tested. Meanwhile, there was no significant differences between the inductions of cell proliferation at 10μM glabridin compared to the other drugs at the concentration where they each induced the maximum estrogenic activity, Fig. 9.

## Conclusions

In conclusion, the results showed that glabridin displayed estrogenic activity in Ishikawa cells via the ER-α-SRC-1-co-activator. The combination study suggests that glabridin could possibly co-treated with 17β-E_2_ as an ERT to induce a higher level of estrogenic activity but without the same intensity of risk on endometrial cancer. Though the results showed that as with 17β-E_2_, glabridin induced dose-dependent increase in both ALP activity and cell proliferation in Ishikawa cells, the combination study results suggested that glabridin could act in a different manner from 17β-E_2_. Therefore it is postulated that glabridin might act differently from 17β-E_2_ via ER-α-independent mechanisms in Ishikawa cells.
