# Correction: Estrogenicity of Glabridin in Ishikawa Cells

**DOI:** 10.1371/journal.pone.0133340

**Published:** 2015-07-17

**Authors:** Melissa Su Wei Poh, Phelim Voon Chen Yong, Navaratnam Viseswaran, Yoke Yin Chia

The following information is missing from the Funding section: This work was supported by Taylor's University through its Postgraduate Research Scholarship Programme to MSWP. The complete, correct funding information is as follows: This work was supported by Exploratory Research Grants Scheme ERGS/1/2012/STG08/TAYLOR/03/2) to YYC, and Fundamental Research Grant Scheme (FRGS/2/2010/SKK/TAYLOR/03/1) to YYC. This work was supported by Taylor's University through its Postgraduate Research Scholarship Programme to MSWP. The funders had no role in study design, data collection and analysis, decision to publish, or preparation of the manuscript.
